# Supplementary material for: Application of Natural Deep Eutectic Solvents in the Extraction of Quercetin from Vegetables
Source: Molecules. 2019 Jun 21;24(12):2300. doi: 10.3390/molecules24122300 (PMC6631864; doi:10.3390/molecules24122300)
Supplement: Supplementary file 1 [file molecules-24-02300-s001.pdf]

Article

Application of Natural Deep Eutectic Solvents in the Extraction of Quercetin from Vegetables

Yunliang Dai and Kyung Ho Row *

Department of Chemistry and Chemical Engineering, Inha University, Incheon 402751, Korea; 22172312@inha.edu

* Correspondence: rowkho@inha.ac.kr; Tel.: +82-32-860-7470

Received: 11 June 2019; Accepted: 20 June 2019; Published: 21 June 2019

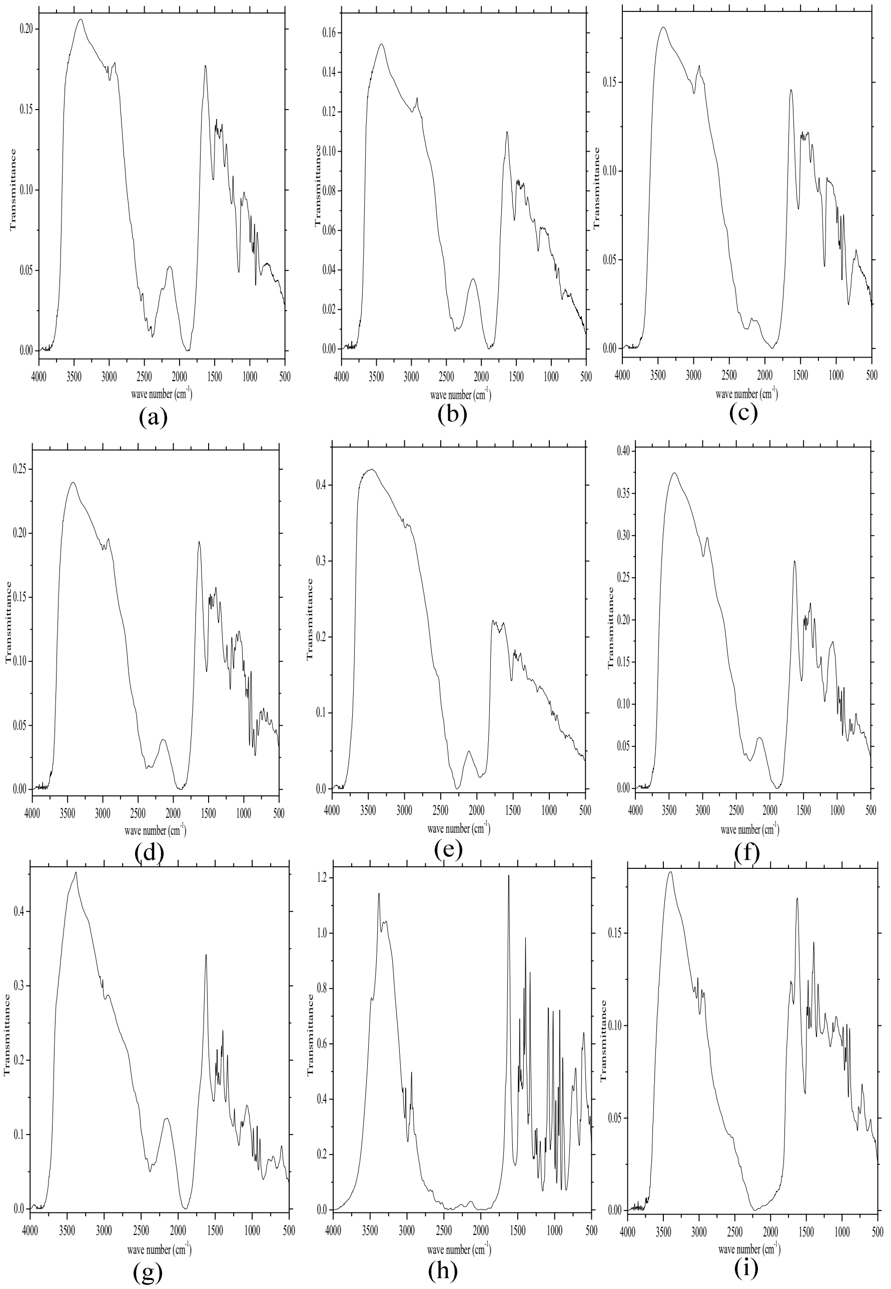

Figure S1. FT-IR spectra of betaine based NADESs. a: Bet-Ery, b: Bet-Rib, c: Bet-Xyl, d: Bet-Fuc, e: Bet-Glul, f: Bet-Man, g: Bet-Gal, h: Bet-Mal, and i: Bet-Glua.

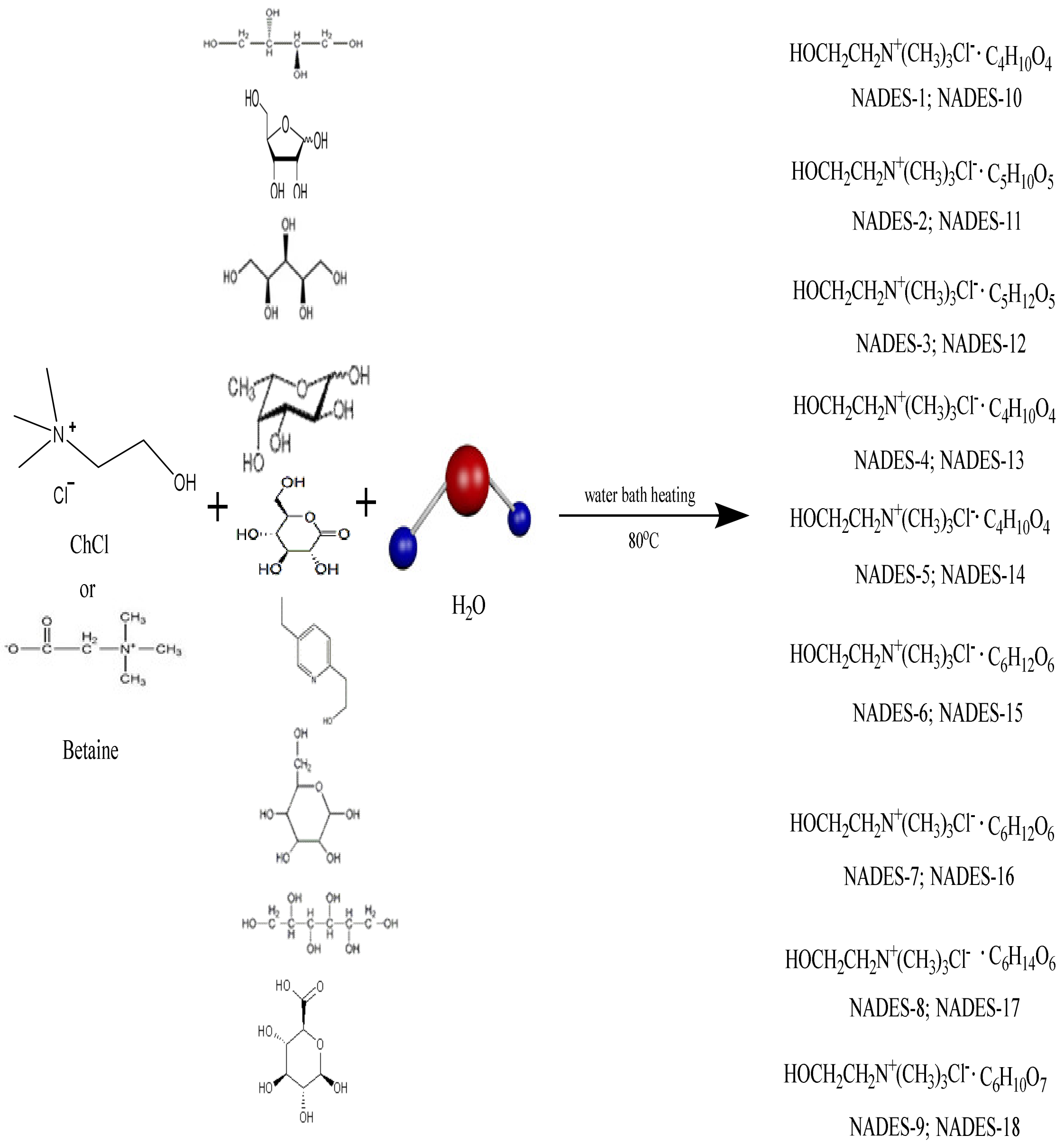

Figure S2. Synthetic protocol of the NADESs.

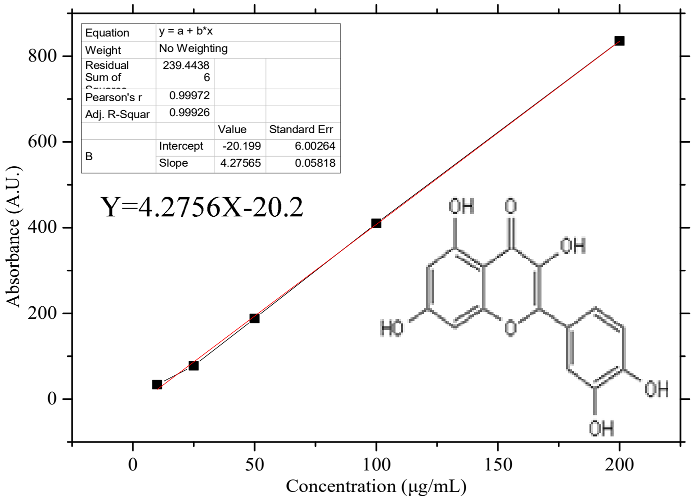

(1) quercetin

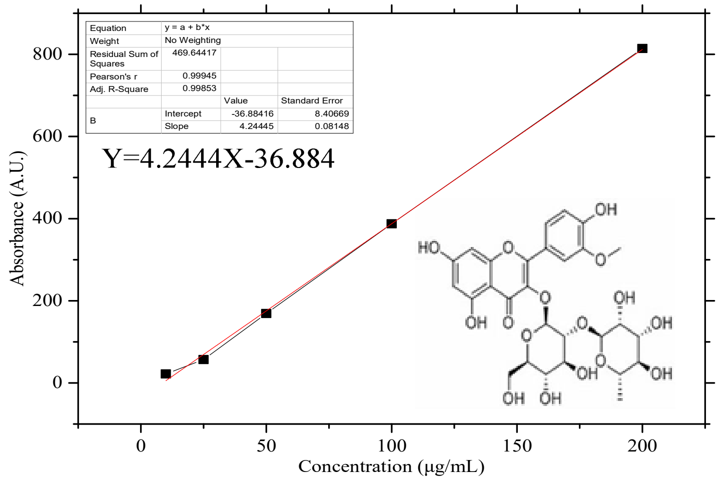

(2) isorhamnetin

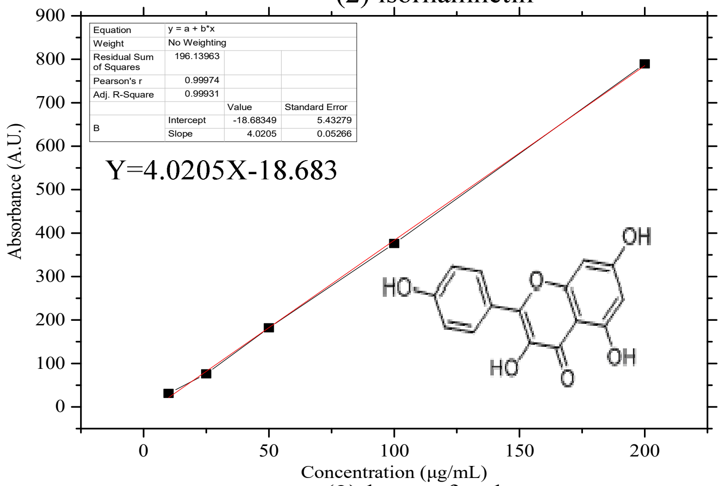

(3) kaempferol

Figure S3. Standard curves and chemical structures of quercetin, isorhamnetin, and kaempferol.

Table S1. Abbreviations of the ChCl-based DESs.

Abbreviations	Component 1	Component 2	Component 3	Molar ratio [a]	Status	Viscosity [c] ($mm^2\ s^{-1}$)
NADES-1	ChCl	meso-Erythritol	H_2O	1:1:0.1	Solid [b]	17.42
NADES-2		D-(-)-Ribose			Liquid	15.64
NADES-3		xylitol			Liquid	16.24
NADES-4		L-(-)-Fucose			Liquid	14.88
NADES-5		D-(+)-Glucono-1, 5-lactone			Liquid	15.72
NADES-6		D-(+)-Mannose			Liquid	20.48
NADES-7		D-(+)-Galactose			Solid	22.16
NADES-8		D-Mannitol			Liquid	13.24
NADES-9		D-Glucuronic acid			Liquid	13.72

a: The best molar ratio, other molar ratios were also studied (including 1:2/2:1/1:1.5) but turned out not to be homogeneous liquids. b: The status of it is solid at room temperature, while at a higher temperature it is liquid. c: The viscosity of it at 25.0 °C (H_2O: 0.7 $mm^2\ s^{-1}$).

Table S2. Abbreviations of the betaine-based DESs.

Abbreviations	Component 1	Component 2	Component 3	Molar ratio	Status	Viscosity ($mm^2\ s^{-1}$)
NADES-10	Bet	meso-Erythritol	H_2O	1:1:1	Solid	10.32
NADES-11		D-(−)-Ribose			Liquid	9.64
NADES-12		xylitol			Liquid	9.72
NADES-13		L-(-)-Fucose			Liquid	10.80
NADES-14		D-(+)-Glucono-1, 5-lactone			Liquid	11.08
NADES-15		D-(+)-Mannose			Liquid	10.24
NADES-16		D-(+)-Galactose			Solid	12.16
NADES-17		D-Mannitol			Solid	12.64
NADES-18		D-Glucuronic acid			Liquid	11.56
